# Factors of COVID-19 Vaccine Perception among Transport Drivers in Singapore: A Cross-Sectional Pilot Study

**DOI:** 10.4269/ajtmh.22-0510

**Published:** 2023-02-06

**Authors:** Evelyn Huizi Lim, Ngan Phoon Fong, Junxiong Pang

**Affiliations:** Saw Swee Hock School of Public Health, National University Health System and National University of Singapore, Singapore

## Abstract

Transport drivers have high risk of exposure to SARS-CoV-2 during COVID-19 pandemic. Vaccines can reduce disease severity. However, COVID-19 vaccine perception among transport drivers is unknown. To identify the key factors influencing vaccine perception of transport drivers in Singapore, a cross-sectional pilot study was conducted. One hundred four completed survey responses were collected between September 2021 and February 2022. Using multivariable logistic regression, education, general vaccine knowledge and attitude, practice of social distancing, misinformation of rare vaccine side effects, and perceiving the pandemic situation to be severe were independently associated with vaccine perception. Despite high vaccination coverage, there were substantial poor vaccine perception, vaccine hesitancy, and unwillingness to take third dose. Vaccination rate may thus not be an accurate reflection of true vaccine acceptance. Communication strategies need to focus on correcting knowledge gaps, instilling collectivist attitudes, and highlighting the importance of vaccination over social distancing to enhance booster uptake rate.

With 511 million cases and 6.2 million deaths worldwide as of May 3, 2022,^1^ COVID-19 has taken a great toll on many healthcare systems and economies. To control the disease, Singapore launched its COVID-19 vaccination program^2^ on December 30, 2020. Drivers and transport workers comprised 18% of work-related transmission cases in Asia. This was attributed to the high frequency of close contact with travelers and the general public.^3^ It was also difficult to trace the source of infection. From July to September 2021, there was a COVID-19 outbreak among bus drivers across several bus interchanges in Singapore.^4^ Although over 95% of eligible drivers had received two doses as of January 2022, about 300 of them had yet to receive the vaccine despite it being made available for almost a year.^5^

Vaccine perception plays a key role in influencing vaccination intent and eventual uptake.^6^ Negative attitudes may result in vaccine hesitancy and hamper global efforts in achieving herd immunity. In Singapore, the SOCRATES cohort study^7^ found that vaccine hesitancy between June and July 2021 was relatively low at 9.9%. Vaccine-specific determinants of vaccine hesitancy were side effects, safety, and rapid development of vaccine. As Singapore adopts a vaccine-driven strategy to transit into an endemic state,^8^ it is imperative to address factors contributing to poor vaccine perception, especially among those with high risks of exposure.

This study aims to investigate COVID-19 vaccine perception and its underlying factors among drivers in Singapore. A cross-sectional study was conducted using an online anonymous questionnaire. Transport drivers aged between 21 and 64 years old were recruited using convenience sampling methods. The primary outcome, vaccine perception, was established using the question “How important do you think getting COVID-19 vaccine is for your health?” Vaccine perception was dichotomized into “good” (very important) versus “poor” (moderately important, a little important, or not at all important). Vaccine hesitancy was determined by the failure of first dose vaccine uptake by June 30, 2021, which is 5 months from the launch of the vaccination program.^2^ Willingness to receive a third dose was dichotomized into “agree” and “do not agree.” Multivariable logistic regression was performed to identify the independent variables associated with vaccine perception. Thematic analysis was performed for the reasons for willingness or unwillingness to receive a third dose. Implied consent was collected online prior to starting the survey. Because the study was anonymous and posed minimal risk, it was exempted from Institutional Review Board review by the Saw Swee Hock School of Public Health Departmental Ethics Review Committee (SSHSPH-150).

One hundred four complete responses out of 155 responses were received. Most respondents were Chinese, Singaporean, and male and reported having at least secondary school education (Table 1). The respondents were largely private drivers, working full-time and exposed to general public daily. Of the respondents, 66.3% (*N* = 69) had good vaccine perception, 6.7% (*N* = 7) were not vaccinated, 20.2% (*N* = 21) were vaccine hesitant, and 71.2% (*N* = 74) were willing to take the third dose Supplemental Table 6).

**Table 1:** Demographic differences between drivers with poor and good vaccine perception

Variables*	Poor vaccine perception (*N* = 35)	Good vaccine perception (*N* = 69)	Total (*N* = 104)	*P* value
Age, years (SD)	44.82 (12.66)	47.92 (10.57)	46.88 (11.35)	0.19
Gender, *n* (%)				
Male	32 (91.4)	65 (94.2)	97 (93.3)	0.45
Female	2 (5.7)	4 (5.8)	6 (5.8)	–
Prefer not to say	1 (2.9)	0 (0.0)	1 (1.0)	–
Ethnicity, *n* (%)				
Chinese	23 (65.7)	56 (81.2)	79 (76.0)	0.104
Malay	7 (20.0)	7 (10.1)	14 (13.5)	–
Indian	3 (8.6)	6 (8.7)	9 (8.7)	–
Other	2 (5.7)	0 (0.0)	2 (1.9)	–
Nationality, *n* (%)				
Singaporean	34 (97.1)	65 (94.2)	99 (95.2)	0.80
Permanent resident	0 (0.0)	2 (2.9)	2 (1.9)	–
Other	1 (2.9)	2 (2.9)	3 (2.9)	–
Education, *n* (%)				
Primary school	6 (17.1)	3 (4.3)	9 (8.7)	0.06
Secondary school and above	29 (82.9)	66 (95.6)	95 (91.3)	–
Marital status, *n* (%)				
Not married	13 (37.1)	20 (29.0)	33 (31.7)	0.40
Household annual income, *n* (%)				
<$40,000	20 (57.1)	41 (66.7)	62 (59.6)	0.71
≥$40,000	15 (42.9)	27 (39.1)	42 (40.4)	–
BMI, *n* (%)†				
Underweight/overweight	27 (77.1)	52 (75.4)	79 (76.0)	0.84
COVID-positive status, *n *(%)				
Self	0 (0.0)	3 (4.3)	3 (2.9)	0.66
Family	3 (8.6)	8 (11.6)	11 (10.6)	0.75
Friends	10 (28.6)	23 (33.3)	33 (31.7)	0.66
Prior infectious disease vaccination (influenza and Dengue) (%)				
Yes	7 (20.0)	27 (39.1)	34 (32.7)	0.05
Chronic diseases, *n* (%)				
Yes	10 (28.6)	29 (42.0)	39 (37.5)	0.42
No	22 (62.9)	34 (49.3)	56 (53.85)	–
Prefer not to say	3 (8.6)	6 (8.7)	9 (8.65)	–
Diabetes	5 (14.3)	16 (23.2)	21 (20.2)	0.29
Hypertension	5 (14.3)	17 (24.6)	22 (21.2)	0.22
Smoking status, *n* (%)				
Smoker	13 (37.1)	19 (27.5)	32 (30.8)	0.32
Occupation, *n* (%)‡				
Public	9 (25.7)	29 (42.0)	38 (36.5)	0.10
Private	26 (74.3)	40 (58.0)	66 (63.5)	–
Work shift type, *n* (%)				
Full-time	26 (74.3)	52 (75.3)	78 (75.0)	0.91
Part-time	9 (25.7)	17 (24.6)	26 (25.0)	–
Number of transport companies, *n* (%)				
One company	24 (68.6)	49 (71.0)	73 (70.2)	0.80
More than one company	11 (31.4)	20 (29.0)	31 (29.8)	–
Exposure to public daily (%)	31 (88.6)	60 (87)	91 (87.5)	0.54

* Univariate analysis was conducted using Student’s *t* test for continuous variables and Pearson’s χ^2^ (if cell count ≥ 5) and Fisher’s exact test (if cell count < 5) for categorical variables.

† Body mass index cutoff values are as follows: underweight < 18 kg/m^2^, 18 kg/m^2^ ≤ normal < 23 kg/m^2^, 23 kg/m^2^ ≤ overweight.

‡ Priority given to likely main occupation if there are more than one selected.

Education, social distancing, general vaccine knowledge and attitude, misinformation of pain redness and swelling as rare side effects, and perceiving the situation to be severe were independently associated with vaccine perception (Table 2). The three vaccination indicators lost their significance after adjustment Supplemental Tables 1–6).

**Table 2:** Univariate and multivariate analysis of factors influencing drivers’ vaccine perception

Variables	Unadjusted model		Adjusted model	
	OR (95% CI)	*P* value	AdjOR (95% CI)	*P* value
Education	4.55 (1.06–19.46)	0.041	17.29 (1.46–204.87)	0.024*
Practice safe distancing at work	3.13 (1.34–7.31)	0.009	10.05 (2.14–47.19)	0.003*
General knowledge on nature of COVID-19 vaccine	2.11 (1.46–3.08)	< 0.001	2.12 (1.14–3.96)	0.018*
The COVID-19 situation in Singapore is severe	3.2 (1.38–7.46)	0.006	2.01 (1.23–3.28)	0.005*
General attitude toward vaccine uptake	1.24 (1.13–1.35)	< 0.001	1.25 (1.10–1.43)	0.001*
Rare side effect of vaccine: pain, redness, and swelling at injection site	0.45 (0.19–1.01)	0.066	0.15 (0.03–0.81)	0.027*
Vaccination status	14.06 (1.62–122.14)	0.016	0.23 (0.01–8.53)	0.425
Vaccine hesitancy	0.22 (0.08–0.61)	0.003	2.40 (0.21–27.73)	0.483
Willingness to receive third dose	6.26 (2.48–15.81)	< 0.001	1.89 (0.39–9.24)	0.432

AdjOR = adjusted odds ratio; OR = odds ratio.

* Significant after adjustment (*P* < 0.05).

The top reasons for taking a third dose were that the booster could provide increased protection for self and family (*N* = 36; 32.4%) or that the vaccine was effective against new variants (*N* = 15; 13.5%); some respondents just took it because it was offered (*N* = 8; 7.2%) (Table 3). The top reasons for unwillingness to take third dose were vaccine ineffectiveness (*N* = 11; 9.9%), vaccine side effects (*N* = 6; 5.4%), and two doses being sufficient (*N* = 5; 4.5%).

**Table 3:** Reasons for willingness or unwillingness to receive third dose

	Themes	Number of responses
Reasons for third dose	Increase protection for self and family	36
	Effective against new variants	15
	Just take it as it is offered	8
	Trust in government and experts	5
	Social responsibility	4
	Regulations, no choice	4
	To remove restrictions and allow activities to resume to normal	4
	Increased risk from working frontline	2
	Strengthen immunity as it will wane off	2
Neutral	No preference	2
	Wait-and-see approach	2
Reasons against third dose	Ineffective	11
	Side effects	6
	Two doses are sufficient	5
	Health reasons	2
Miscellaneous	Miscellaneous group	3

Similar to Ruiz and Bell’s findings,^9^ education was found to have the strongest effect on drivers’ vaccine perception. Education was commonly found to be positively associated with factors influencing positive health outcomes.^10^ According to Larson,^11^ however, education could influence vaccine acceptance in both directions because underlying political and cultural factors could shape overall vaccine acceptance. In the drivers’ context, low literacy may generate knowledge gaps about vaccines, which indirectly gives rise to negative attitudes. Although it could be difficult to enhance education, outreach programs could focus on closing the specific knowledge gaps identified.^12^

Poor vaccine knowledge was associated with poor vaccine perception. This corroborates Ruiz and Bell’s findings.^9^ One identified knowledge gap was the misconception that COVID-19 vaccines were ineffective. This was also one of the top reasons for unwillingness to receive the third dose, whereas the opposite reason was given for willingness to receive the third dose. Another misconception that was associated with poor vaccine perception is that pain, redness, and swelling were rare side effects of vaccine. These could have negatively affected their perception if mismatch of expectations arose due to misinformation and deterred them from taking future booster doses. One respondent was unwilling to receive a third dose because he was misinformed that the booster dose was not approved by the WHO. Another respondent had asked for evidence to prove that booster dose was necessary. It is thus pertinent for health authorities to address such knowledge gaps.^13^ Through improving the knowledge of drivers, their perceived benefits of vaccination, which is part of the Health Belief Model, could also be improved. This sets the foundation for building confidence in COVID-19 vaccines.

Perceiving the COVID-19 situation to be severe but not perceiving susceptibility was positively associated with vaccine perception. This concurs with others studies.^10^^,^^14^ By emphasizing the severity of the situation in vaccination messages, drivers would perceive the disease to be severe and become more wary. The WHO,^15^ for instance, has highlighted that the Omicron variant could cause severe disease in vulnerable individuals. Because the majority of respondents had chronic diseases, it is possible for them to suffer from more severe symptoms if infected. Vaccination messages should thus focus on perceived severity.

Collectivist attitude, which highlights the importance of community and conformity to social norms,^16^ is another critical aspect of vaccine perception.^8^ This was demonstrated by the overall high vaccine acceptance in Asian countries compared with European countries.^17^ Drivers with good vaccine perception tended to want to protect their friends and family from the disease. This was also the top reason for the drivers’ willingness to take the third dose. This could be especially so if their loved ones are vulnerable. Shah et al.^18^ had found that Singaporeans residing with children below 5 years and with elderly persons had higher vaccine intent. Drivers with good vaccine perception also felt it was their social responsibility to be vaccinated and help return life to normal. Messages that emphasize social responsibility and protection of loved ones may thus improve vaccine perception. Also underlying the collectivist attitude is the trust in health authorities and vaccine manufacturers. This corroborates the findings by Wong et al.^19^ that vaccine acceptance was associated with government’s recommendations. Our qualitative finding also found that respondents who were willing to receive the third dose trusted the government to act in their best interest. All eight respondents who indicated they had received the booster had also received their first dose within 4 months. This indicated strong underlying trust in the government. To gain trust, governments and manufacturers need to be transparent about the benefits and risks of the vaccines to their best knowledge.

Vaccine hesitancy among the drivers (20.2%) in our study was higher than the general population (9.9%)^7^ and primary healthcare workers^20^ (5.1%) in Singapore. Compared with healthcare workers, greater effort may be needed to shift drivers’ vaccine perception because they may have lower health literacy and could be more susceptible to misinformation and misconception. Because vaccine perception was not independently associated with the key vaccination indicators, other factors could have greater influence over these vaccine indicators. One possible factor is the use of regulation to drive vaccination rates. Drivers are required to be “fully vaccinated” to continue working.^5^ There could also be other factors that were not covered within the scope of study. Furthermore, due to the small study size, there could be insufficient statistical power to identify independent associations with these indicators. Conducting the study online could result in biased estimates due to the under-representation of drivers with limited internet access and self-selection. Our findings may thus not be generalizable to the whole Singaporean driver population.

Although high vaccine coverage could be driven by regulation, there were substantial proportions of drivers with poor vaccine perception, vaccine hesitancy, and unwillingness to receive the third dose. Despite the lack of association with the vaccination indicators, it is still critical to address poor vaccine perception if vaccination is to a remain long-term strategy in the battle against COVID-19 and other vaccine-preventable diseases as part of our pandemic preparedness. These could be achieved through open communication to address the gaps identified in knowledge, attitudes, and practices.

## Supplemental files


Supplemental materials
