# Supplementary material for: Factors of COVID-19 Vaccine Perception among Transport Drivers in Singapore: A Cross-Sectional Pilot Study
Source: Am J Trop Med Hyg. 2023 Feb 6;108(3):588–91. doi: 10.4269/ajtmh.22-0510 (PMC9978561; doi:10.4269/ajtmh.22-0510)
Supplement: Supplementary file 1 [file tpmd220510.SD1.pdf]

Supplementary materials:

Supplementary Table 1: Knowledge scores on COVID-19 and vaccine.

**Category**	**Knowledge Type (Total Score)**	**Poor Vaccine Perception (n=35)**	**Good Vaccine Perception (n=69)**	**Total (N=104)**	**Odds Ratio (OR)**	**95% Confidence Interval**	**P-value**
COVID-19 disease (A)	Symptoms of COVID-19 disease (9)	5.43 (1.74)	5.68 (1.42)	5.60 (1.53)	1.12	0.85-1.46	0.43
	Transmission of COVID-19 (9)	7.28 (1.32)	7.46 (1.50)	7.40 (1.44)	1.09	0.82-1.44	0.55
	General knowledge on nature of COVID-19 (4)	2.97 (0.86)	2.81 (0.83)	2.87 (0.84)	0.79	0.48-1.31	0.36
	Total COVID-19 disease knowledge score (22)	15.69 (2.67)	15.96 (2.26)	15.87 (2.63)	1.03	0.89-1.21	0.62
COVID-19 vaccine (B)	Rare side effects of COVID-19 vaccine (9)	5.51 (1.74)	5.85 (2.12)	5.74 (2.21)	1.07	0.89-1.29	0.46
	Meaning of vaccine Efficacy (1)	0.14 (0.36)	0.26 (0.44)	0.22 (0.42)	2.11	0.71-6.29	0.18
	Efficacy of COVID-19 vaccine brands (3)	1.21 (1.21)	1.81 (1.07)	1.61 (1.15)	1.61	1.11-2.34	**0.01**

Supplementary Table 1: Knowledge scores on COVID-19 and vaccine.

	Eligibility criteria for COVID-19 vaccine (10)	6.77 (1.86)	7.30 (1.34)	7.13 (1.55)	1.25	0.96-1.62	0.10
	General knowledge on nature of COVID-19 vaccine (8)	5.26 (1.65)	6.55 (1.01)	6.12 (1.40)	2.11	1.46-3.08	**<0.001**
	Total COVID-19 vaccine knowledge score (31)	18.88 (4.19)	21.78 (3.43)	20.81 (3.93)	1.24	1.09-1.41	**0.001**
Total Knowledge Score	A+B (53)	34.57 (4.97)	37.74 (4.84)	36.67 (5.09)	1.14	1.04-1.25	**0.004**

Supplementary Table 2. Breakdown of COVID-19 knowledge components

**Knowledge Items**	**Poor Vaccine Perception (n=35)**	**Good Vaccine Perception (n=69)**	**Total (N=104)**	**Odds Ratio (OR)**	**95% Confidence Interval**	**P-value**
**General knowledge on nature of COVID-19 vaccine**						
*Vaccine can can reduce the severity of the symptoms of COVID-19 disease if infected.	24 (68.6%)	61 (88.4%)	85 (81.7%)	3.49	1.25-9.75	**0.02**
Less than 55% of COVID-19 vaccine uptake is required to achieve herd immunity.	1 (2.86%)	0 (0.0%)	1 (1.0%)	-	-	-
COVID-19 vaccines are not effective against the new COVID-19 strains.	10 (28.6%)	8 (11.6%)	18 (17.3%)	0.33	0.12-0.93	**0.04**
Additional booster dose of vaccine is not effective against the new COVID-19 strains.	10 (28.6%)	6 (8.7%)	16 (15.4%)	0.24	0.078-0.724	**0.01**
*The protective effect of COVID-19 is at least 6 months.	10 (28.6%)	33 (47.8%)	43 (41.4%)	2.29	0.95-5.48	0.06
I do not need to be vaccinated If I have been infected with COVID-19 previously.	11 (31.4%)	6 (8.7%)	17 (16.4%)	0.21	0.069-0.62	**0.01**
COVID-19 vaccine could give me COVID-19 disease.	3 (8.6%)	1 (1.5%)	4 (3.9%)	0.16	0.02-1.57	0.12
*I still qualify for other COVID-19 vaccines even if I am allergic to one particular brand.	10 (28.6%)	34 (49.3%)	44 (42.3%)	2.43	1.02-5.81	**0.05**
*: Correct Answer						

Supplementary Table 2. Breakdown of COVID-19 knowledge components

**COVID-19 Vaccine Eligibility Criteria**						
History of severe allergic reaction	27 (77.1%)	53 (76.8%)	80 (76.9%)	0.98	0.37-2.58	0.97
*Severely weakened immune system	18 (51.4%)	35 (50.7%)	53 (51.0%)	0.97	0.43-2.19	0.95
*Very low platelet counts of less than 50,000	14 (40.0%)	23 (33.3%)	37 (35.4%)	0.75	0.32-1.74	0.50
Diabetes	5 (14.3%)	2 (2.9%)	7 (6.7%)	0.18	0.33-0.98	**0.05**
High Cholesterol	4 (11.4%)	2 (2.9%)	6 (5.8%)	0.23	0.04-1.33	0.10
High Blood Pressure	6 (17.1%)	2 (2.9%)	8 (7.7%)	0.14	0.03-0.76	**0.02**
G6PD Deficiency	6 (17.1%)	10 (14.5%)	16 (15.4%)	0.82	0.27-2.47	0.72
Taking TCM (Traditional Chinese Medicine)	3 (8.6%)	3 (4.4%)	6 (5.8%)	0.48	0.09-2.54	0.39
Undergoing Kidney Dialysis	13 (37.1%)	21 (30.4%)	34 (32.7%)	0.74	0.31-1.74	0.49
Received another vaccine for a different disease	11 (31.4%)	13 (18.8%)	24 (23.1%)	0.51	0.20-1.29	0.15
*: Correct Answer						
**Rare Side Effects of Vaccine**						
Pain, Redness and swelling at injection site	16 (45.7%)	19 (27.5%)	35 (33.7%)	0.45	0.19-1.01	0.07
Vomitting	7 (20.0%)	12 (17.4%)	19 (18.3%)	0.84	0.30-2.37	0.75
Fever and chills	10 (28.6%)	221 (30.4%)	31 (29.8%)	1.09	0.45-2.68	0.84
*Infection/injury of heart muscles	35 (42.9%)	27 (39.1%)	42 (40.4%)	0.86	0.38-1.96	0.71
*Blood clots	15 (42.9%)	35 (50.7%)	50 (48.1%)	1.37	0.61-3.11	0.45

Supplementary Table 2. Breakdown of COVID-19 knowledge components

Headache, muscle pain and joint pain	11 (31.4%)	18 (26.1%)	29 (27.9%)	0.77	0.32-1.88	0.57
*Severe allergic reaction	16 (45.7%)	33 (47.8%)	49 (47.12%)	1.09	0.48-2.46	0.84
Lymph node swelling at neck and arms	8 (22.9%)	18 (26.1%)	26 (25.5%)	1.19	0.46-3.09	0.72
A feeling of constant tiredness or weakness	11 (31.4%)	17 (24.6%)	28 (26.9%)	0.71	0.29-1.75	0.46
*: Correct Answer						

Supplementary Table 3: Perceived Severity and Susceptibility

**Attitude towards COVID-19**	**Poor Vaccine Perception (n=)**	**Good Vaccine Perception (n=69)**	**Total (N=104)**	**Odds Ratio (OR)**	**95% Confidence Interval**	**P-value**
**Perceived Severity**						
The COVID-19 situation in Singapore is severe.	15 (40.0%)	47 (68.1%)	61 (58.7%)	3.2	1.38-7.46	**0.007**
The emergence of mutant COVID-19 strains is of grave concern.	18 (51.4%)	56 (81.2%)	74 (71.2%)	4.07	1.66-9.97	**0.002**
I am afraid of contracting COVID-19 infection.	19 (54.3%)	47 (68.1%)	66 (63.5%)	1.8	0.78-4.15	0.168
I am afraid of transmitting the SAR-COV2 virus to my family and friends.	26 (74.3%)	63 (91.3%)	89 (85.6%)	3.63	1.17-11.2	**0.025**
I will not be able to provide and care for my family if I am infected with COVID-19.	25 (71.4%)	59 (85.5%)	84 (80.8%)	2.36	0.87-7.37	0.09
I will be avoided by others if I am infected with COVID-19.	21 (60.0%)	54 (78.3%)	75 (72.1%)	2.4	0.99-5.82	0.053
**Perceived Susceptibility**						
I am generally healthy therefore I am not vulnerable to COVID-19 infection and its complications.	10 (28.6%)	26 (37.7%)	36 (34.6%)	1.51	0.63-3.65	0.358
The nature of my work puts me at risk of COVID-19 infection.	27 (77.1%)	58 (84.06)	85 (81.7%)	1.56	0.56-4.32	0.391

Supplementary Table 4: Attitude scores on COVID-19 and vaccines

**Category**	**Attitudes**	**Poor Vaccine Perception (n=35)**	**Good Vaccine Perception (n=69)**	**Total (N=104)**	**Odds Ratio (OR)**	**95% Confidence Interval**	**P-value**
COVID-19 disease (A)	Importance of Safety Management Measures	65.65 (9.79)	72.06 (13.82)	69.90 (12.92)	1.03	1.01-1.07	**0.02**
COVID-19 vaccine (B)	General attitude towards vaccine uptake	27.14 (8.04)	36.86 (5.88)	33.59 (8.09)	1.24	1.13-1.35	**<0.001**
	Number of cues to action	31.49 (10.16)	39.29 (8.99)	36.66 (10.06)	1.08	1.04-1.14	**0.001**
	Number of vaccine concerns	2.02 (1.15)	1.72 (1.06)	1.83 (1.09)	0.78	0.54-1.12	0.18

Supplementary Table 5. Breakdown of attitude components

Attitude Items	Poor Vaccine Perception (n=35)	Good Vaccine Perception (n=69)	Total (N=104)	Odds Ratio (OR)	95% Confidence Interval	P-value
**Importance of the following safety management measures**						
Mask wearing	31 (88.6%)	63 (91.3%)	94 (90.4%)	1.35	0.36-5.15	0.656
Handwashing/Sanitising	32 (91.4%)	66 (95.7%)	98 (94.2%)	2.06	0.39-10.79	0.391
Safe Distancing	29 (82.9%)	60 (87.0%)	89 (85.3%)	1.38	0.45-4.24	0.575
Avoiding crowded areas	29 (82.9%)	61 (82.9%)	90 (86.5%)	1.58	0.50-4.97	0.436
Reducing the number and size of social gatherings	25 (71.4%)	60 (87.0%)	85 (81.7%)	1.38	0.45-4.24	0.575
Disinfecting surfacecs	31 (88.6%)	64 (92.8%)	95 (91.4%)	1.65	0.41-6.58	0.477
Travel Restrictions	20 (57.1%)	56 (81.2%)	76 (73.8%)	3.23	1.31-7.96	**0.011***
Quarantine for those awaiting swab test results	24 (68.6%)	60 (87.0%)	84 (80.8%)	3.06	1.12-8.31	**0.029***
Monitoring of temperature	13 (37.1%)	49 (71.0%)	62 (59.6%)	4.15	1.75-9.80	**0.001***
Installing shield between driver and passenger seats	13 (37.1%)	41 (59.4%)	54 (51.9%)	2.47	1.07-5.72	**0.034***
Opening vehicle windows	14 (40.0%)	51 (73.9%)	65 (62.5%)	4.25	1.79-10.08	**0.001***
Avoiding going to work when having runny nose /sore throat/ cough	28 (80.0%)	66 (95.7%)	94 (90.4%)	5.5	1.32-22.82	**0.019***
**Attitude towards vaccine uptake**						
Social Responsibility	22 (62.9%)	68 (98.6%)	90 (86.5%)	40.18	4.97-324.86	**0.001***
Protect friends and family	23 (65.7%)	65 (94.2%)	88 (84.6%)	8.48	2.48-28.93	**0.001***

COVID-19 vaccination will allow life to return to normal	16 (45.7%)	59 (85.5%)	75 (72.1%)	7.01	2.73-18.01	**<0.001***
Trust in manufacturers	9 (25.7%)	53 (76.8%)	62 (59.6%)	9.57	3.73-24.54	**<0.001***
Trust in health authorities	15 (42.9%)	63 (91.3%)	78 (75.0%)	14	4.79-40.90	**<0.001***
COVID-19 vaccination can reduce the severity of the symptoms of COVID-19	22 (62.9%)	66 (95.7%)	88 (64.6%)	13	3.39-49.89	**<0.001***
Will still need to continue to follow prevention measures even after vaccination.	28 (80.0%)	66 (95.7%)	94 (90.4%)	14	4.79-40.90	**<0.001***
**Cues to action**						
Government Recommendation	21 (60.0%)	60 (87.0%)	81 (77.9%)	4.44	1.68-11.77	**0.003***
Employer Recommendation	17 (48.6%)	43 (62.3%)	60 (57.7%)	1.75	0.77-3.99	0.18
Healthcare Workers Recommendation	17 (48.6%)	60 (87.0%)	77 (74.0%)	7.06	2.69-18.52	**<0.001***
Family Members Recommendation	13 (37.1%)	53 (76.8%)	66 (63.5%)	5.61	2.31-13.58	**<0.001***
Friends Recommendation	13 (37.1%)	49 (71.0%)	62 (59.2%)	4.15	1.75-9.80	**0.001***
There are more people taking it	11 (31.4%)	51 (73.9%)	62 (59.6%)	6.18	2.53-15.10	**<0.001***
Can travel without restrictions if vaccinated	14 (40.0%)	43 (62.3%)	57 (54.8%)	2.48	1.08-5.71	**0.03***
**Vaccine Preference**						
mRNA vaccine	16 (45.7%)	50 (72.5%)	66 (63.5%)	-	-	**0.001***

Inactivated virus vaccine	1 (2.9%)	7 (10.1%)	8 (7.69%)	2.24	0.26-19.61	
No preference	15 (42.9%)	12 (17.4%)	27 (26.0%)	0.26	0.10-0.66	
Not interested to take COVID-19 vaccine	3 (8.6%)	0 (0.0%)	3 (2.9%)	1	-	
**Concerns regarding COVID-19 vaccine**						
Short term side-effects	5 (14.3%)	17 (24.6%)	22 (21.2%)	1.96	0.66-5.86	0.23
Long term side effects	21 (60.0%)	38 (55.1%)	59 (56.7%)	0.82	0.36-1.87	0.63
The vaccine is effective enough	13 (37.1%)	16 (23.19%)	29 (27.9%)	0.51	0.21-1.24	0.14
Not been enough testing of vaccines	14 (40.0%)	21 (30.4%)	35 (33.7%)	0.66	0.28-1.53	0.33
Using a newer platform of vaccine	10 (28.6%)	15 (21.7%)	25 (24.0%)	0.69	0.27-1.76	0.44
Do not qualify to receive one yet	0 (0.0%)	0 (0.0%)	0 (0.0%)	-	-	-
Not given preferred vaccine	0 (0.0%)	0 (0.0%)	0 (0.0%)	0.75	0.12-4.71	0.76
Difficult to get appointment	0 (0.0%)	2 (2.9%)	2 (1.9%)	-	-	-
Difficult to take time away from work and caring responsibilities	1 (2.9%)	3 (4.4%)	4 (3.9%)	1.55	0.15-15.42	0.71
Locations of vaccination centres are inconvenient	0 (0.0%)	1 (1.5%)	1 (1.0%)	-	-	-
Medical Reasons	1 (2.9%)	1 (1.5%)	2 (1.9%)	0.5	0.03-8.24	0.63
Other concerns	4 (11.4%)	2 (2.9%)	6 (5.8%)	0.23	0.04-1.33	0.10

Supplementary Table 6. Univariate Analysis of Vaccination Indicators

**Vaccination Information**	**Poor Vaccine Perception (n=35)**	**Good Vaccine Perception (n=69)**	**Total (N=104)**	**Odds Ratio (OR)**	**95% Confidence Interval**	**P-value**
**Vaccination Status**						
Not Vaccinated	6 (17.1%)	1 (1.5%)	7 (6.7%)	14.06	1.62-122.14	0.016*
Vaccinated	29 (82.9%)	68 (98.6%)	97 (93.27%)			
**Vaccine Hesitancy**						
Acceptant	22 (62.9%)	61 (88.4%)	83 (79.8%)	0.22	0.08-0.61	0.002*
Hesitant	13 (37.1%)	8 (11.6%)	21 (20.2%)			
**Willingness to take third dose**						
Willing	16 (45.7%)	58 (84.1%)	74 (71.2%)	6.26	2.48-15.81	**<0.001***
